# Influence of host-specific and locally isolated multi-strain probiotics on piglet performance, mortality, inflammatory response, and gut microbiome

**DOI:** 10.5713/ab.24.0556

**Published:** 2024-10-28

**Authors:** Katatikarn Sahatsanon, Panneepa Sivapirunthep, Korawan Sringarm, Chaiwat Arjin, Patipan Hnokaew, Kamon Chaweewan, Chanporn Chaosap

**Affiliations:** 1 Doctoral Program in Innovative Tropical Agriculture, Department of Agricultural Education, School of Industrial Education and Technology, King Mongkut’s Institute of Technology Ladkrabang, Bangkok 10520, Thailand; 2 Department of Agricultural Education, School of Industrial Education and Technology, King Mongkut’s Institute of Technology Ladkrabang, Bangkok 10520, Thailand; 3 Department of Animal and Aquatic Sciences, Faculty of Agriculture, Chiang Mai University, Chiang Mai 50200, Thailand; 4 Office of Research Administration, Chiang Mai University, Chiang Mai 50200, Thailand; 5 Bureau of Animal Husbandry and Genetic Improvement, Department of Livestock Development, Pathum Thani 12000, Thailand

**Keywords:** *Lactobacillus* spp., Microbial Community, Pro-Inflammatory Cytokines, Weaned Pigs

## Abstract

**Objective:**

This study aimed to assess the impact of host-specific and locally isolated multi-strain probiotics on piglet performance, mortality, inflammatory responses, and gut microbiome.

**Methods:**

A total of 52 piglet litters-34 from Landrace sows and 18 from Large White sows-were allocated to two groups: a control group and a multi-strain probiotic group. The probiotic group comprised seven strains of lactic acid bacteria (MLAB): *Lactobacillus brevis, Lactobacillus reuteri, Lactobacillus paraplantarum, Lactococcus lactis, Lactobacillus pentosus, Weissella cibaria*, and *Pediococcus pentosaceus*. Each strain was included in equal concentrations, resulting in a final liquid mixture containing 10^9^ colony forming units/mL. The MLAB group received the probiotics orally starting from 7 days of age until weaning at four weeks. Following weaning, supplementation continued via feed spraying for an additional four weeks.

**Results:**

MLAB supplementation did not significantly affect piglet performance but showed a trend towards reducing the mortality rate (p = 0.06). It influenced the inflammatory response by upregulating the expression of anti-inflammatory cytokines interleukin (IL)-4 and IL-10 (p<0.05). Microbial community analysis indicated that MLAB supplementation increased both microbial diversity (Simpson index: p = 0.06) and species richness (Chao1 index: p = 0.02). Piglets receiving MLAB had a significantly higher abundance of the phylum *Firmicutes* (p<0.01) compared to the control group, while the abundance of the phylum *Bacteroidota* was markedly reduced (p<0.01). In addition, the relative abundance of the bacterial genera *Prevotellaceae*_NK3B31 (p<0.01) and *Chlamydia* (p = 0.03) was lower in the MLAB group.

**Conclusion:**

Overall, these results suggest that while MLAB supplementation does not directly improve piglet growth performance, it has the potential to improve immune function and promote a healthier gut microbiota in weaning piglets, which could ultimately reduce mortality rates.

## INTRODUCTION

In pig production, various factors such as age, nutrition, and environment have a significant impact on animal health and productivity [1]. Among these factors, piglets are particularly vulnerable during the transition from suckling to weaning. This critical period can induce stress, leading to changes in the function and morphology of the small intestine. These changes can impair nutrient absorption and digestion, compromise the intestinal barrier and lead to reduced feed intake, increased feed conversion ratio (FCR), weight loss, diarrhea and possibly death [2,3]. To mitigate these effects, various supplements, including probiotics, are added to piglet feed to improve health and reduce stress [4,5]. Probiotics are beneficial live microorganisms that provide health benefits to the host when ingested in sufficient quantities [6]. They are used in animal production to improve gut health, immune function, and overall productivity [1,5]. Probiotics also produce antimicrobial compounds such as bacteriocin, diacetyl, hydrogen peroxide, and organic acids [7].

Lactic acid bacteria (LAB) are gram-positive, non-spore-forming bacteria that can be rod-shaped or coccoid. They are tolerant to low pH values and mainly produce lactic acid by breaking down carbohydrates [7,8]. LAB genera include *Lactobacillus*, *Pediococcus*, *Enterococcus*, *Leuconostoc*, *Lactococcus*, *Streptococcus*, and *Weissella*, with *Lactobacillus* being the largest genus [7]. *Lactobacillus*, which is often used as a probiotic in animal feed, lowers the pH of the digestive tract by producing lactic acid and thus inhibits the growth of pathogens [9,10].

Single-strain probiotics have been shown to improve feed intake, average daily gain (ADG), and FCR [8,11]. Probiotics can reduce diarrhea and improve anti-inflammatory cytokines like interleukin (IL)-10 and transforming growth factor (TGF)-β [8]. Multiple-strain probiotics have shown benefits in terms of better adhesion, inhibition of gut pathogens, and improvement of ADG, feed per gain, immune system, and microbiota composition in piglets [10,12]. Although commercial probiotic products are widely available, species-specific and locally adapted microorganisms are crucial for improving pig health. Porcine-specific probiotics have been shown to enhance growth and provide protection against pathogens such as Escherichia coli and Salmonella, particularly in newborn and weaned pigs. These probiotics achieve their effects by inhibiting pathogen adhesion, reducing bacterial translocation, and producing inhibitory molecules—benefits that are not typically observed with non-porcine-specific probiotics [13,14].

Although numerous studies have been conducted on probiotics, there is still a need for the discovery of new strains with beneficial properties, as the efficacy of probiotics is strain-specific. This study is a continuation of a previous study funded by the Thailand Institute of Scientific and Technological Research (TISTR), which focused on screening potential probiotic LAB for the swine industry [15]. Seven lactic acid bacteria strains were identified as potential probiotics: *Lactobacillus brevis*, *Lactobacillus reuteri*, *Lactobacillus paraplantarum*, *Lactococcus lactis*, *Lactobacillus pentosus*, *Weissella cibaria*, and *Pediococcus pentosaceus*. All strains were isolated from healthy pigs in Thailand and selected for their probiotic properties, including their ability to adapt to the gastrointestinal tract of pigs and their species-specific safety.

The objective of this study was to examine how seven multi-lactic acid bacterial (MLAB) strains impact performance, mortality, inflammation-related gene expression, and the gut microbiome in piglets.

## MATERIALS AND METHODS

### Animal ethics

The Ethics Committee of the Department of Livestock Development, Ministry of Agriculture and Cooperatives of Thailand (DLD036/64) approved all animal studies.

### Multi-lactic acid bacterial probiotic preparation

The probiotics used in this study were obtained from TISTR. The LAB strains were isolated from pigs reared in Thailand. The isolation of the LAB strains and the preparation of the mixed MLAB inoculum are described in detail in Supplementary Materials. The specific bacterial strains included *Lactobacillus brevis*, *Lactobacillus reuteri*, *Lactobacillus paraplantarum*, *Lactococcus lactis*, *Lactobacillus pentosus*, *Weissella cibaria*, and *Pediococcus pentosaceus*. The probiotic formulation consisted of seven LAB strains, each at a similar concentration, which were mixed in liquid form to achieve a final concentration of 10^9^ colony forming units (CFU)/mL.

### Rearing methods, diets, and probiotics used

The study was conducted at the Swine Research and Development Center in Pak Chong District, Nakhon Ratchasima, Thailand.

A total of 52 farrowing litters, each litter of mixed-sex piglets, were divided into two groups: the control group and the MLAB group. The control group consisted of 9 litters from Large White (LW) sows and 16 litters from Landrace (LR) sows, while the MLAB group consisted of 9 litters from LW sows and 18 litters from LR sows, for a total of 25 litters in the control group and 27 litters in the MLAB group. Litter sizes ranged from 8 to 17 piglets in the control group and 8 to 16 piglets in the MLAB group, with both groups having an average of 11 piglets per litter. The mode was 9 piglets per litter for both groups, while the median was 10 piglets for the control group and 11 piglets for the MLAB group. The farrowing pens with slatted floors measured 2 m×2.2 m. Each pen allocated 0.6 m×2.2 m for the sow and 1.4 m×2.2 m for the piglets and feeding area. The sow had a feeding space of 30 cm×30.5 cm×17 cm, while the piglets used a round feeder with a diameter of 25 cm and a depth of 6 cm. A single water nipple was provided for the sow. After birth, the piglets’ umbilical cords and tails were cut, their teeth were clipped, and their ears were tagged for identification. Each piglet received a 2 mL injection of iron solution. All male piglets were castrated. After the piglets in both groups ingested colostrum and transitioned to sow’s milk, only the piglets in the MLAB group received 2 mL of the MLAB probiotic solution orally every three days to minimize handling stress, starting from 7 days of age until weaning at four weeks. All piglets were provided with a standard creep feed containing 22% protein from 7 days of age until weaning. The sows were fed a commercial diet with a protein content of 16% and had ad libitum access to clean water.

After weaning, each litter of piglets was moved to a nursery pen, one litter per pen. Each pen was 2.5 m×2.5 m and had a height of 95 cm. The number of weaned piglets per pen varied between 6 and 15 in the control group and between 4 and 16 in the MLAB group, with an average of 10 piglets per pen in both groups. The mode was 8 piglets per pen and the median was 9 piglets per pen for the control group, while both the mode and the median were 10 piglets per pen for the MLAB group. Both groups were fed a commercial diet containing 20% crude protein. The MLAB group received an additional 5 mL of MLAB solution per piglet daily for four weeks. The piglets were fed twice a day, at 7 a.m. and 4 p.m. To ensure consistent dosing, 5 mL of MLAB solution per 100 g of feed was sprayed and prepared in the morning meal, calculated based on the total number of piglets in each litter. The feed was then thoroughly mixed to ensure uniform distribution, allowing each piglet equal access. During the evening feeding, feed was provided ad libitum throughout the four-week experiment. Vaccinations against classical swine fever and foot-and-mouth disease were administered at 6 and 7 weeks of age respectively. Sows and piglets were reared in an open housing system from birth to 8 weeks of age.

### Data and sample collection

Birth weight and weights at 3, 4 and 8 weeks of age were recorded for each piglet to calculate the ADG for each litter. In addition, the number of piglets born alive and the number of deaths in each litter were recorded from birth to 8 weeks of age.

At 8 weeks of age, 15 piglets from each group (9 from LR litters and 6 from LW litters) were sampled for gene expression and microbiome analysis. Piglets were anesthetized prior to euthanasia by intravenous injection of 1 mL thiopental (sodium pentothal) followed by intravenous administration of saturated magnesium sulfate (MgSO4) to induce cardiac arrest. Tissue samples were taken from the Peyer’s patches (mesenteric lymph nodes) in the ileum of 15 piglets from each group (9 from LR litters and 6 from LW litters) to evaluate gene expression. These samples were first stored in liquid nitrogen and then kept at −80°C until analysis of inflammatory gene expression. In addition, fecal samples were randomly collected from 12 of the euthanized piglets (7 from LR litters and 5 from LW litters) in each group. These samples were also stored at −80°C for later DNA extraction, which was used to analyze the amount and composition of microorganisms in the feces.

### Inflammatory gene expression analysis

#### RNA extraction and cDNA synthesis

After extracting total RNA from each Peyer’s patch sample, a deoxyribonuclease was employed to eliminate genomic DNA impurities. cDNA was generated in a final volume of 15 μL using random primers and Moloney murine leukemia virus reverse transcriptase, following the manufacturer’s instructions (Promega ImProm-II Reverse Transcription, Promega, Madison, WI, USA). The primers used in this work [16,17] are shown in Table 1.

#### Quantitative real-time polymerase chain reaction

For gene expression analysis, first-strand cDNA samples were diluted 1:5. The reaction mixture, prepared in duplicate on a 96-well plate for a Bio-Rad CFX96 system (Hercules, CA, USA), included 3.5 μL of cDNA, 0.4 μL of each primer, and 5 μL of SYBR Green Universal PCR Master Mix (SensiFastTM SYBR, Bioline, London, UK). The thermal protocol involved polymerase activation at 95°C for 2 minutes, followed by 40 cycles of 95°C for 5 seconds and 60°C for 15 seconds. Fluorescence was monitored in real time, and specificity was confirmed with a melting curve analysis. Relative expression was calculated using the 2^−ΔΔCT^ method [18], normalizing to glyceraldehyde-3-phosphate dehydrogenase (GAPDH).

### Microbiome analysis

#### DNA extraction and amplicon sequencing

Total DNA was isolated from colon feces using the QIAamp Fast DNA Stool Mini Kit (QIAGEN, Hilden, Germany) in accordance with the manufacturer’s instructions. DNA quality was determined using 2% agarose gel electrophoresis and measured with a NanoDrop 2000C spectrophotometer (Thermo Scientific, Waltham, MA, USA). Polymerase chain reaction (PCR) amplification of the V3–V4 region of the 16S rRNA genes was conducted using primers 341F (5′-CCTAYGGGRBGCASCAG-3′) and 806R (5′-GGACTACNNGGGTATCTAAT-3′) [19]. The PCR cycling conditions were as follows: 1 minute of initial denaturation at 98°C, followed by 30 cycles of denaturation at 98°C for 10 seconds, annealing at 50°C for 30 seconds, and elongation at 72°C for 5 minutes, with a final elongation step at 72°C for 10 minutes. The PCR mixture consisted of 15 μL Phusion High Fidelity PCR Master Mix (New England Biolabs, Ipswich, MA, USA), 0.2 μL forward primer, 0.2 μL reverse primer, and 10 ng template DNA. The PCR products were purified using a Qiagen Gel Extraction Kit (Qiagen, Dusseldorf, Germany) and quantified using a Qubit 2.0 fluorometer (Thermo-Fisher, Waltham, MA, USA). Finally, partial 16 S rRNA sequencing was performed by next-generation sequencing utilizing the Illumina NovaSeq 6000 platform (Illumina Inc., San Diego, CA, USA), which generated thousands of 250 bp paired-end reads.

#### Bioinformatic analysis

After sequencing, the raw data were classified by sample using an index sequence, and each sample was assigned paired-end FASTQ files. The quality of the raw FASTQ files was checked using FastQC version 0.12.1 software. Raw sequences were then demultiplexed and barcodes and adaptor sequences were removed using Cutadapt software version 4.5. The Quantitative Insights Into Microbial Ecology 2 version 2023.9 (QIIME2) pipeline was used to analyze the obtained raw sequences, and the trimmed sequences were denoised and merged using the DADA2 plugin within QIIME2 for amplicon sequence variants (ASVs). The taxonomic assignment of the 16S rRNA sequences was performed using the Silva 138 99% taxonomy classifier. The absolute abundance of ASVs was normalized to the sequence number of the sample with the fewest sequences. The normalized data were then used for alpha and beta diversity analysis.

### Statistical analysis

A randomized complete block design (RCBD) was used as the experimental design. The breed of the piglets was used as a block, namely the LR and LW breeds. The experimental model was as follows:

Y_ijk_ = μ + Ai + Bj + €ijk
Y_ijk_ = Observations derived
μ = Overall mean excluding experimental influences
A_i_ = Influence due to diet factor i (where i represents the control and MLAB groups)
B_j_ = Influence due to breed as a block effect j (where j represents LR and LW piglets)
€_ijk_ = Random error of the observation

Analysis of variance (ANOVA) was used to compare the differences between the means of the traits studied, including piglet production performance and expression of inflammatory genes. The SPSS version 29 program was used to analyze the data.

To compare the number of piglets that died during the 8-week rearing period between the control and MLAB groups, the data were analyzed using the statistical method of the chi-square goodness-of-fit test.

For the analysis of the gut microbiota, the alpha diversity indices (Simpson and Chao1 indices) were calculated using the QIIME2 software. The statistical differences between the control and MLAB groups in alpha diversity were determined with the Mann–Whitney U test using R software version 4.3.1.

Permutative multivariate ANOVA (PERMANOVA) (Vegan package, function adonis) was used to analyze beta diversity reflecting differences in bacterial community composition between groups based on Bray–Curtis dissimilarity distance [20]. Relationships between samples were visualized using principal coordinate analysis (PCoA) in R software.

Differences in microbial taxa at phylum and genus level between groups were statistically determined using the Mann–Whitney U test in R software.

### Data availability

The 16 S sequencing data for the control and MLAB groups were uploaded to the NCBI Sequence Read Archive (SRA) (https://www.ncbi.nlm.nih.gov/sra) under BioProject ID: PRJNA1133190.

## RESULTS

In the present study, the administration of MLAB to piglets did not significantly affect body weight or ADG (p>0.05), as shown in Table 2. Chi-square analysis revealed a tendency toward a lower number of dead piglets in the MLAB group compared to the control group (p = 0.06), with a mortality ratio of 49 out of 280 piglets in the control group compared to 32 out of 289 piglets in the MLAB group (Table 3).

Table 4 illustrates the effects of MLAB supplementation on the expression of inflammatory genes in 8-week-old piglets. No statistically significant differences were observed in the relative gene expression of pro-inflammatory cytokines, including IL-8, IL-12p35, IL-12p40, interferon (IFN)-γ and tumor necrosis factor (TNF)-α, and anti-inflammatory cytokines such as porcine beta-defensin (pBD)-2 and cyclooxygenase (COX)-2 between the control and MLAB groups (p>0.05). However, the MLAB group had higher relative gene expression levels of IL-4 and IL-10 than the control group (p<0.05), with IL-4 levels of 0.60 versus 0.39 and IL-10 levels of 1.58 versus 0.96.

**Figure 1:**
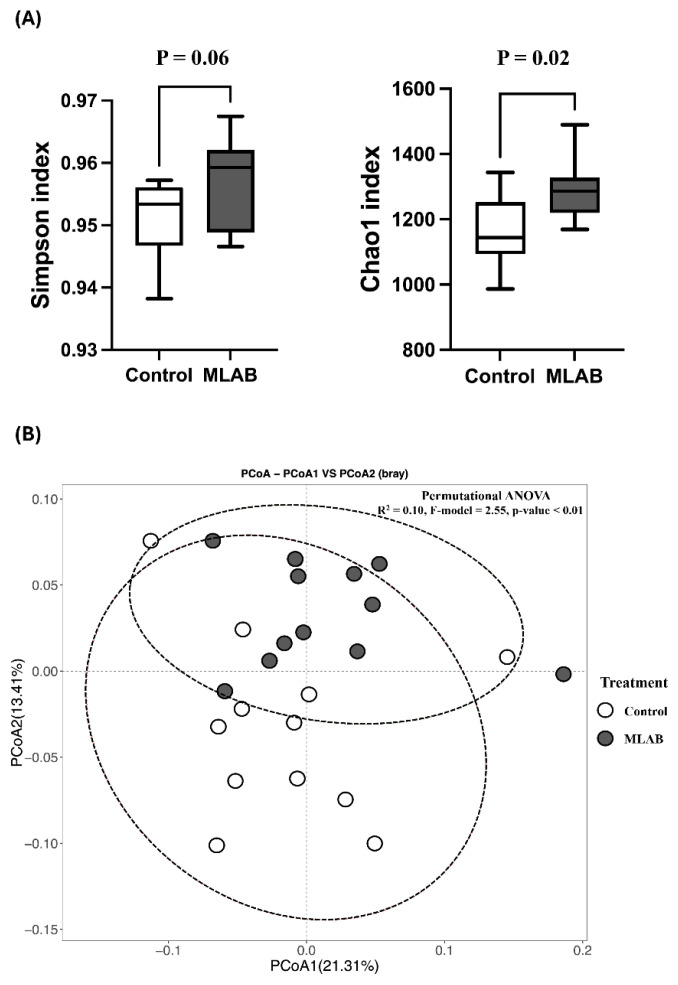
The analysis of the gut microbiota. (A) Diversity and richness of microorganisms in piglet feces between control and multi-lactic acid bacteria (MLAB) by Simpson index and Chao1 index; (B) analysis of beta diversity by principal coordinates analysis (PCoA) in the feces of piglets between control and multi-lactic acid bacteria (MLAB) groups. ANOVA, analysis of variance. The p-value (P) indicates a statistically significant difference when p<0.05.

Alpha diversity indicates the diversity and richness of microorganisms, as shown in Figure 1A. The Simpson index of microbial diversity in the piglets’ feces showed a higher trend in the MLAB group (p = 0.06). The species richness of microorganisms measured with the Chao1 index was also significantly higher in the MLAB group than in the control group (p = 0.02).

Analysis of the beta diversity of bacterial ASVs based on the Bray-Curtis dissimilarity matrix showed that the composition of the microbial community was clearly separated between the control and MLAB groups. PCoA analysis revealed that beta diversity in the feces accounted for 21.31% of the variation between the control and MLAB groups in PC1 and 13.41% of the variation in PC2. In addition, PERMANOVA analysis showed that the probiotic contributed significantly to the differences in microbial community composition (p<0.01) (Figure 1B).

**Figure 2:**
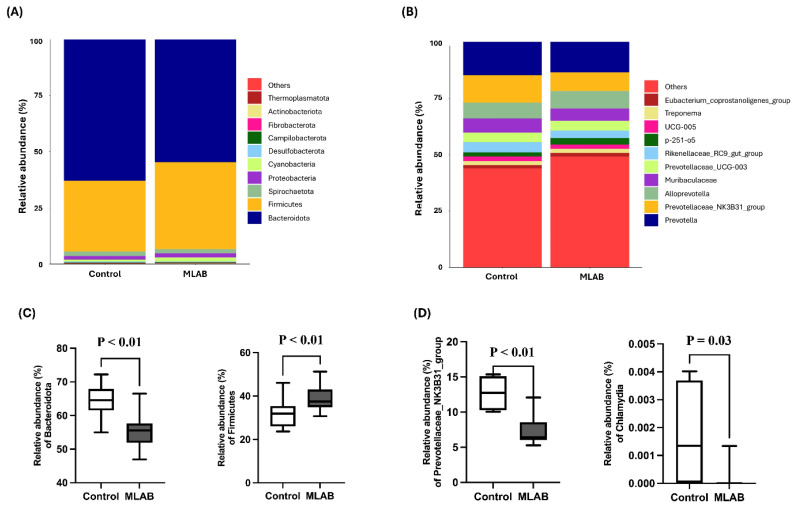
Differences in microbial taxa between the control groups and multi-lactic acid bacteria (MLAB). Histogram of the relative abundance of microbiota in piglet feces at (A) phylum level and (B) genus level. Differences in the microbial community in piglet feces by Mann-Whitney U-test at (C) phylum level and (D) genus level. The p-value (P) indicates a statistically significant difference when p<0.05.

The relative abundance of the 10 most significant microbial communities in piglet feces for the control and MLAB groups, at both the phylum and genus levels, is shown in Figures 2A, 2B. The major microbial communities in the control group were the phyla *Bacteroidota* (62.90%) compared to 54.61% in the MLAB group, *Firmicutes* (31.51% vs. 38.74%), *Spirochaetota* (2.02% vs. 1.86%), Proteobacteria (1.56% vs. 1.92%), and Cyanobacteria (0.93% vs. 1.80%). The other phyla, each accounting for less than 1%, were *Desulfobacterota*, *Campylobacterota*, *Fibrobacterota*, *Actinobacteriota*, and *Thermoplasmatota* (Figure 2A). At the genus level, the microbial community composition of the control group compared to the MLAB group included *Prevotella* (17.65% vs. 16.42%), *Prevotellaceae*_NK3B31_group (14.66% vs. 10.07%), *Alloprevotella* (8.50% vs. 9.38%), *Muribaculaceae* (7.47% vs. 6.73%), *Prevotellaceae*_UCG-003 (5.16% vs. 5.15%), Rikenellaceae_RC9_gut_group (5.33% vs. 4.02%), p-251-o5 (2.23% vs. 3.63%), UCG-005 (2.50% vs. 2.21%), Treponema (2.02% vs. 2.21%), and *Eubacterium_coprostanoligenes*_group (1.84% vs. 2.08%) (Figure 2B). The Mann–Whitney U test revealed significant differences in the relative of bacterial populations at the phylum *Firmicutes* and *Bacteroidota* (p<0.01), as shown in Figure 2C. At the genus level, 36 genera showed significant differences between the control and MLAB groups (Supplementary Material 1). The 10 genera with the highest values were *Prevotellaceae*_NK3B31_group, *Parabacteroides*, UCG.002, *Anaerovibrio*, *Ruminococcus*, *Clostridium_sensu_stricto*_1, *Agathobacter*, *Sphaerochaeta*, *Lachnospiraceae*_NK4A136_group, and *Faecalibacterium*. Among these 36 genera, the control group had a higher *Prevotellaceae*_NK3B31_group than the MLAB group (p<0.01). *Chlamydia*, which was identified as an indicator of pathogenicity, was less prevalent in the MLAB group (p = 0.03) (Figure 2D).

## DISCUSSION

Ideally, a probiotic supplement for pigs should promote both growth and overall health. In the present study, piglets supplemented with MLAB did not exhibit a significant increase in body weight or ADG during the first four weeks. It is well established that piglet weight gain during the first three weeks is largely determined by their intake of colostrum, transitional milk, and mature milk, as well as the microbial communities present in these milk types [21]. One of the predominant bacterial phyla found in sow milk is *Firmicutes* [21], which is the same phylum as the MLAB used in this study. Therefore, the addition of these microorganisms may not significantly affect the population of endogenous microorganisms, thereby explaining the absence of an effect of MLAB on piglet performance compared to the control group.

Probiotics can enhance performance in pigs by improving nutrient digestion. For example, *Lactobacillus* spp. produce lactic acid and proteolytic enzymes that support growth. Research has shown that both single-strain [22,23] and multi-strain [10,11,24] probiotic supplements can increase growth rates in post-weaning pigs. However, some studies have reported no growth-promoting effects from single-strain probiotic supplementation [8]. Lähteinen et al [25] found that a mixture of six *Lactobacillus* species-including *Lactobacillus amylovorus*, *Lactobacillus mucosae*, *Lactobacillus salivarius*, *Lactobacillus johnsonii*, and *Lactobacillus reuteri*-at a concentration of 10^10^ cells/mL in the diets of post-weaning piglets had no significant effect on ADG. Similarly, the probiotic mixture used in this study, which comprised five strains of *Lactobacillus*, one strain of *Weissella*, and one strain of *Pediococcus*, yielded comparable results.

The variability in the effects of probiotics on piglet performance may stem from several influencing factors. First, the dynamic and established characteristics of the gut microbiome can hinder the ability of probiotic strains to effectively colonize and compete [26]. Second, environmental factors-including genetics, management practices, hygiene, diet, and overall health-also play a crucial role [25,26]. Additionally, the effectiveness of probiotics in enhancing growth performance is influenced by variables such as the specific strain used, the dosage administered, the age at which supplementation begins, and the duration of the feeding trial [10,11,22–25]. Duddeck et al [27] reported that supplementing with high levels of probiotics does not necessarily improve growth performance in piglets.

In the present study, the number of dead piglets in the MLAB group tended to be lower than in the control group. These results are consistent with those of Wang et al [8] who observed that weaned piglets fed *Lactobacillus plantarum* PFM105 had a lower mortality rate (0.00%) than those fed antibiotics (2.08%) and the control group (4.17%). The lower mortality rate in the probiotic group may be due to the ability of *Lactobacillus* to colonize and adhere to the epithelium of the digestive tract by forming a protective barrier against pathogenic microorganisms [28]. In addition, Inatomi et al [29] reported a significant decrease in mortality among piglets naturally infected with Porcine Epidemic Diarrhea virus when supplemented with mixed probiotics (*Bacillus subtilis*, *Clostridium butyricum*, and *Enterococcus faecium*), showing a 41% lower mortality rate compared to the control group (91%). They suggested that probiotic supplementation helps prevent the attachment of pathogenic microorganisms to intestinal mucosal cells by competing with the pathogens and potentially promoting the production of bacteriocins.

Environmental and dietary changes during weaning can trigger stress leading to intestinal problems such as diarrhea, decreased growth rate, and reduced feed intake [3]. This study found that supplementation with MLAB increased the expression of the anti-inflammatory genes IL-4 and IL-10. This effect may be attributed to the protective role of probiotics against pathogen-induced intestinal inflammation [2]. IL-10 is of crucial importance for immune homeostasis as it inhibits pro-inflammatory cytokines [8]. In our study, higher IL-10 gene expression was observed in the MLAB group compared to the control group, which could explain the lower mortality of the MLAB group. In agreement with these results, Wang et al [8] reported increased expression of IL-10, TGF-β, and IL-2 in piglets supplemented with *Lactobacillus plantarum* PFM105. Lähteinen et al [25] reported that gene expression of IFN-α, IL-4, and IL-6 was increased in piglets and gene expression of TNF and IL-8 was decreased in piglets fed mixed *Lactobacillus* spp. (with *Lactobacillus amylovorus*, *Lactobacillus mucosae*, *Lactobacillus salivarius*, *Lactobacillus johnsonii*, and *Lactobacillus reuteri*). In addition, Tan et al [30] demonstrated that IL-4 and IL-10 produced by T helper (Th2) cells which suppressed the production of the pro-inflammatory cytokines IFN-γ and IL-2 produce from Th1 cells. In addition, Sarkar et al [12] found that anti-inflammatory IL-4 levels were higher in piglets receiving a mixed probiotic containing *Enterococcus faecalis*, *Clostridium butyricum*, *Bacillus mesentericus*, and *Bacillus coagulans* than in the control group, which is consistent with this study in which piglets in the MLAB group had higher IL-4 gene expression and correspondingly lower mortality. Probiotic supplements increased Th2-related cytokines such as IL-4 and thus improved immune function [31]. Yu et al [11] reported that dietary *Lactobacillus* can form a protective barrier in the digestive tract and stimulate white blood cells, thereby improving overall immunity.

To study the gut microbiome of piglets, microbial diversity and uniformity were assessed using the Simpson index, while species richness was evaluated using the Chao1 index. The Simpson index reflects both diversity and uniformity of microorganisms, with higher values indicating greater species uniformity [32]. The Chao1 index, on the other hand, measures species richness [33]. In this study, the Simpson index tended to be higher in the MLAB-supplemented groups, while the Chao1 index was significantly higher than in the control group. According to Shin et al [1] and Janczyk et al [34], the administration of probiotics in the first 7 days after birth and at weaning has a positive effect on the gut microbiota by changing the intestinal environment. Several studies also suggest that the administration of probiotics can help to restore the balance of the bacterial community in weaned piglets [1,34,35].

In this study, the relative abundance of microbiota in piglets showed that the MLAB-fed group had a higher abundance of the phylum *Firmicutes* and a lower abundance of *Bacteroidota*. The increased *Firmicutes* population in the feces of the MLAB-treated piglets could be due to the high concentration of *Lactobacillus* strains in the MLAB supplement, since *Lactobacillus* belongs to the phylum *Firmicutes* [35]. These results are in agreement with those of Shin et al [1], who observed that piglets treated with *Lactobacillus plantarum* JDFM LP1 had higher *Firmicutes* and lower *Bacteroidota* levels.

At the genus level, the *Prevotellaceae*_NK3B31 group, which belongs to the phylum *Bacteroidota*, was more abundant in the control group than in the MLAB-fed group. This difference could be related to the higher stress level in the control group. The *Prevotellaceae*_NK3B31 group, which is commonly found in the gastrointestinal tract of nursery pigs, was positively correlated with cortisol levels in weaned pigs (r = 0.76, p = 0.03), suggesting an association with post-weaning stress [5]. In addition, infections caused by *Chlamydia*ceae in pigs can lead to a range of health problems, e.g. pneumonia, enteritis, weak piglets, increased neonatal mortality and reproductive disorders in sows [36]. In this study, the population of the genus *Chlamydia* was reduced in the MLAB-fed group, which could contribute to a better overall health status of the piglets, as lower *Chlamydia* levels are associated with fewer health complications. In this study, there was no significant differences in the abundance of *Lactobacillus* genus in the feces between two groups. This suggests that the addition of *Lactobacillus* may not significantly impact the microbial balance, as *Lactobacillus* is a natural component of the piglets’ intestinal flora and may already be present at saturation levels within the gut ecosystem [26,37].

## CONCLUSION

MLAB supplementation had no significant effect on body weight or ADG of weaning piglets, but showed a trend towards lower mortality. The MLAB group showed higher expression of the anti-inflammatory cytokines IL-4 and IL-10 and greater microbial diversity. MLAB supplementation also increased the abundance of beneficial *Firmicutes*, decreased *Bacteroidetes* and reduced the pathogenic genus *Chlamydia*. Although MLAB did not directly improve growth performance, it could improve the immune response and promote a healthier gut microbiota, which in turn could reduce mortality.

## IMPLICATION

The results of this study suggest that supplementation with locally isolated multi-strain probiotics is less effective in promoting piglet growth performance than previously hypothesized. This emphasizes the need for further research to evaluate the efficacy of local probiotic strains compared to well-studied global strains. Although growth performance did not improve significantly, the trends in immune response and gut microbiota composition suggest potential health benefits, warranting further investigation of microbial interactions to improve overall animal health.

## Figures and Tables

**Table 1:** The primers used in this study

Gene	Primer sequence (5′ to 3′)	Reference
*IL-4*	Forward: GGTCTGCTTACTGGCATGTACC Reverse: CTCCATGCACGAGTTCTTTCTC	[16]
*IL-8*	Forward: AGAACTGAGAAGCAACAACAACAG Reverse: CACAGGAATGAGGCATAGATGTAG	
*IL-10*	Forward: GATATCAAGGAGCACGTGAACTC Reverse: GAGCTTGCTAAAGGCACTCTTC	
*IL-12p35*	Forward: TGCAGGCTCTGAATTTCAAC Reverse: CACGAATTCTGAAGGCATGA	
*IL-12p40*	Forward: CTTCATCAGGGACATCATCAAAC Reverse: GGTCCGTGAAGAGTTTATCTTTCT	
*pBD-2*	Forward: CCGACCACTACATATGTGCCAAGA Reverse: TGCCACTGTAACAGGTCCCTTCAA	
*IFN-γ*	Forward: AGGTTCCTAAATGGTAGCTCTGGG Reverse: AGTTCACTGATGGCTTTGCGCT	
*TNF-α*	Forward: CACTGACCACCACCAAGAATTGGA Reverse: CATTCCAGATGTCCCAGGTTGCAT	
*COX-2*	Forward: AAGCGAGGACCAGCTTTCACCAAA Reverse: GCGCAGTTTATGCTGTCTCTCCAA	

*GAPDH*	Forward: TCACTGCCACCCAGAAGA Reverse: TACCAGGAAATGAGCTTGAC	[17]

IL, interleukin; pBD-2, porcine beta defensin-2; IFN-γ, interferon-gamma; TNF- α, tumor necrosis factor-alpha; COX-2, cyclooxygenase-2; GAPDH, glyceraldehyde-3-phosphate dehydrogenase.

**Table 2:** Effect of the supplementation of probiotics on the performance of piglets from birth to 8 weeks of age

Items	Treatment		p-value

	Control (EMM±SE)	MLAB (EMM±SE)	
n (litters)	25	27	
Initial body weight (kg)	1.37	1.43±0.04	0.27
Final body weight (kg)	19.12	18.35±1.11	0.37
Average daily gain (g/d)			
Birth-3 weeks	218.05±8.69	222.57±8.44	0.70
Birth-4 weeks	202.88±8.03	208.19±7.80	0.63
4–8 weeks	453.64±39.97	395.89±38.86	0.29
Birth - 8 weeks	328.26±20.23	302.04±19.67	0.35

Control, piglet diets without addition of MLAB; EMM, estimate marginal means; SE, standard error; MLAB, addition of multi-lactic acid bacteria in piglet diets.

**Table 3:** Effect of the supplementation of probiotics on mortality rate of piglets

Items	Treatment	

	Control	MLAB
Birth to 4 weeks		
Total piglets (n)	280	289
Death piglets (n)	33	22
Chi-square	2.20	
p-value	0.14	
4 to 8 weeks		
Total piglets (n)	247	267
Death piglets (n)	16	10
Chi-square	1.39	
p-value	0.24	
Birth to 8 weeks		
Total piglets (n)	280	289
Death piglets (n)	49	32
Chi-square	3.57	
p-value	0.06	

Control, piglet diets without addition of MLAB; MLAB, addition of multi-lactic acid bacteria in piglet diets.

**Table 4:** Effect of probiotics supplement in diet on relative gene expression (fold change)^1)^ of piglets

Genes	Treatment		p-value
	Control (EMM±SE)	MLAB (EMM±SE)	
*IL-4*	0.39±0.04^b^	0.60±0.07^a^	0.03
*IL-8*	0.55±0.11	0.35±0.05	0.15
*IL-10*	0.96±0.18^b^	1.58±0.17^a^	0.02
*IL-12p35*	0.41±0.10	0.50±0.11	0.58
*IL-12p40*	0.76±0.18	0.73±0.11	0.88
*pBD-2*	0.77±0.11	0.67±0.08	0.49
*IFN-γ*	0.61±0.12	0.52±0.05	0.51
*TNF-α*	0.77±0.17	0.67±0.08	0.60
*COX-2*	0.34±0.06	0.52±0.10	0.13

Control, piglet diets without addition of MLAB; EMM, estimate marginal means; SE, standard error; IL, interleukin; pBD-2, porcine beta defensin-2; IFN-γ, interferon-gamma; TNF-α, tumor necrosis factor; COX-2, cyclooxygenase-2; MLAB, addition of multi-lactic acid bacteria in piglet diets.

1) Relative expression was determined by normalizing the Ct value of the target gene with the Ct value of glyceraldehyde-3-phosphate dehydrogenase (GAPDH), the reference gene, using the 2^−ΔΔCT^ method.

a,b Different superscript letters in the same row indicate a significant difference (p<0.05).

## SUPPLEMENTARY MATERIAL

**Supplementary material 1.** Multi-lactic acid baterial probiotics
