# Supplementary material for: Influence of host-specific and locally isolated multi-strain probiotics on piglet performance, mortality, inflammatory response, and gut microbiome
Source: Anim Biosci. 2024 Oct 28;38(4):717–27. doi: 10.5713/ab.24.0556 (PMC11917409; doi:10.5713/ab.24.0556)
Supplement: Supplementary file 1 [file ab-24-0556-Supplementary-Table-1.pdf]

Supplementary Material S1.

Multi-lactic acid baterial probiotics

Isolation of lactic acid bacteria (LAB)

Forty fecal samples from healthy piglets (30 days old) and parent breeders were collected from swine production farm in the north-east region of Thailand. Each sample weighing about 1 g was serially diluted. 0.1 ml of appropriate dilutions were placed on the surface of prepared MRS supplemented by 0.004 % of bromocresol purple. After anaerobic cultivation at 37ºC for 48-hour, 5 single colonies of each sample with different yellow haloes and morphologies were randomly selected and re-streak twice on MRS agar to obtain pure isolates. The selected isolates were firstly measured cell morphology, gram staining, motility, catalase reaction, and exopolysaccharide production. Two hundred and seventeen isolates were selected and studied further based on probiotic characteristics. A total of 7 isolated LAB was selected as they were not potential pathogenic bacteria. Besides, they exhibited antimicrobial activity against specific pathogens and tolerance to some antimicrobial substances. These isolates were identified by using the API 50 CHL identification kit and confirmed by using the 16S rRNA sequence. Results showed that the potential probiotic LAB were *Lactobacillus reuteri*, *L. brevis*, *L. pentosus*, *L. paraplantarum*. *Lactococcus lactis*, *Wessella cibaria* and *Pediococcus pentosaceus* (data not shown) [1,2].

Preparation of mixed LAB inoculum

A small amount of each LAB from the cultured plate was transferred into a 20-ml MRS both bottles. The liquid culture was incubated at 37°C until cell growth was observed, usually after 24 to 48 hours. The cell suspension was then propagated further in liquid media for 18 hours to ensure that all the cells were harvested from the early stationary phase. Each bacterial suspension was harvested by centrifugation at 3500 rpm for 10 min. The bacterial pellet was washed twice with sterile 0.85% NaCl solution and resuspended in sterile 0.85% NaCl. The concentrations of each LAB were adjusted to 10^{10} cfu/ml in a total volume of 1000 ml. Seven suspensions of each LAB were mixed together to obtain mixed LAB suspension. After that, mixed LAB suspension was mixed with a commercial diet to obtain a final mixed LAB concentration of 1 × 10^8 cfu/kg [3].

References

1. Nuñez IM, Verni MC, Argañaraz Martinez FE, et al. Novel Lactic Acid Bacteria Strains from Regional Peppers with Health-Promoting Potential. Fermentation 2024;10. https://doi.org/10.3390/fermentation10040209
2. Saman P, Chaiongkarn A, Moonmangmee S, et al. Research and development of effective mixed microorganism for piglet production: Institute of Scientific and Technological Research (TISTR), Pathum Thani, Thailand.; 2022. Report No.: 1.

3. Yang F, Hou C, Zeng X, Qiao S. The use of lactic Acid bacteria as a probiotic in Swine diets. Pathogens 2015;4:34-45. http://doi.org/10.3390/pathogens4010034

Taxon	Control	MLAB	SEM	p-value
Prevotellaceae_NK3B31_group	12.7157	7.3728	0.7631	0.000
Parabacteroides	1.8304	1.3420	0.1144	0.028
UCG.002	1.4753	0.9442	0.1657	0.045
Anaerovibrio	0.6774	1.3946	0.1184	0.002
Ruminococcus	0.6755	1.0955	0.0942	0.012
Clostridium_sensu_stricto_1	0.3643	0.5583	0.0562	0.033
Agathobacter	0.3594	0.9224	0.0888	0.000
Sphaerochaeta	0.3431	0.0474	0.0587	0.001
Lachnospiraceae_NK4A136_group	0.3041	0.7477	0.1626	0.046
Faecalibacterium	0.2572	0.3537	0.0379	0.005
Subdoligranulum	0.1831	0.2991	0.0336	0.012
Eubacterium_xylanophilum_group	0.1477	0.2509	0.0241	0.008
Oribacterium	0.1352	0.2167	0.0260	0.040
Dorea	0.1226	0.2187	0.0405	0.018
Roseburia	0.1121	0.2867	0.0323	0.002
Fibrobacter	0.0987	0.1488	0.0141	0.040
Lachnospiraceae_UCG.004	0.0958	0.1627	0.0139	0.011
Romboutsia	0.0821	0.1468	0.0163	0.028
Lachnospiraceae_UCG.010	0.0597	0.0928	0.0081	0.046
Oxalobacter	0.0431	0.0251	0.0043	0.009
Catenibacterium	0.0386	0.0402	0.0122	0.026
Lachnospiraceae_NK4B4_group	0.0340	0.0800	0.0096	0.007
Bacteroides_pectinophilus_group	0.0335	0.0782	0.0102	0.021
Asteroleplasma	0.0258	0.0090	0.0046	0.046
Anaerobiospirillum	0.0209	0.0042	0.0055	0.014
Lachnospiraceae_FCS020_group	0.0193	0.0092	0.0021	0.027
Actinobacillus	0.0177	0.0955	0.0244	0.042
Family_XIII_UCG.001	0.0177	0.0284	0.0027	0.043
Eubacterium_hallii_group	0.0145	0.0256	0.0034	0.018

GCA.900066575	0.0128	0.0236	0.0023	0.021
Eubacterium_ventriosum_group	0.0068	0.0138	0.0017	0.042
Allisonella	0.0045	0.0173	0.0031	0.016
CAG.56	0.0038	0.0081	0.0019	0.023
Lachnospiraceae_UCG.001	0.0017	0.0113	0.0029	0.044
Chlamydia	0.0011	0.0001	0.0003	0.029
Caproiciproducens	0.0000	0.0021	0.0004	0.001
